# The *p*revention *o*f *d*elirium in *e*lderly surgical patients with obstructive *s*leep *a*pnea (PODESA): a randomized controlled trial

**DOI:** 10.1186/s12871-022-01831-1

**Published:** 2022-09-14

**Authors:** Jean Wong, Helen R. Doherty, Mandeep Singh, Stephen Choi, Naveed Siddiqui, David Lam, Nishanthi Liyanage, George Tomlinson, Frances Chung

**Affiliations:** 1 Department of Anesthesiology and Pain Medicine, Toronto Western Hospital, University Health Network, University of Toronto, Toronto, ON Canada; 2 Department of Anesthesiology and Pain Medicine, Women’s College Hospital, Toronto, ON Canada; 3 Institute of Medical Science, Faculty of Medicine, University of Toronto, Toronto, ON Canada; 4 Department of Anesthesia, Sunnybrook Health Sciences Centre, University of Toronto, Toronto, ON Canada; 5 Department of Anesthesia, Mount Sinai Hospital, University of Toronto, Toronto, ON Canada; 6 Institute of Health Policy, Management and Evaluation, Department of Medicine, University of Toronto, Toronto, ON M5G 2C4 Canada

**Keywords:** Obstructive sleep apnea, Delirium, Older adults, Elderly, Positive airway pressure, Home sleep apnea tests, Postoperative

## Abstract

**Background:**

Obstructive sleep apnea (OSA) is associated with neurocognitive impairment – a known risk factor for postoperative delirium. However, it is unclear whether OSA increases the risk of postoperative delirium and whether treatment is protective. The objectives of this study were to identify OSA with a home sleep apnea test (HSAT) and to determine whether auto-titrating positive airway pressure (APAP) reduces postoperative delirium in older adults with newly diagnosed OSA undergoing elective hip or knee arthroplasty.

**Methods:**

We conducted a multi-centre, randomized controlled trial at three academic hospitals in Canada. Research ethics board approval was obtained from the participating sites and informed consent was obtained from participants. Inclusion criteria were patients who were $\ge 6$0 years and scheduled for elective hip or knee replacement. Patients with a STOP-Bang score of ≥ 3 had a HSAT. Patients were defined as having OSA if the apnea–hypopnea index was ≥ 10/h. These patients were randomized 1:1 to either: 1) APAP for 72 h postoperatively or until discharge, or 2) routine care after surgery. The primary outcome was postoperative delirium, assessed twice daily with the Confusion Assessment Method for 72 h or until discharge or by chart review. The secondary outcome measures included length of stay, and perioperative complications occurring within 30 days after surgery.

**Results:**

Of 549 recruited patients, 474 completed a HSAT. A total of 234 patients with newly diagnosed OSA were randomized. The mean age was 68.2 (6.2) years and 58.6% were male. Analysis was performed on 220 patients. In total, 2.7% (6/220) patients developed delirium after surgery: 4.4% (5/114) patients in the routine care group, and 0.9% (1/106) patients in the treatment group (*P* = 0.21). The mean length of stay for the APAP vs. the routine care group was 2.9 (2.9) days vs. 3.5 (4.5) days (*P* = 0.24). On postoperative night 1, 53.5% of patients used APAP for 4 h/night or more, this decreased to 43.5% on night 2, and 24.6% on night 3. There was no difference in intraoperative and postoperative complications between the two groups.

**Conclusions:**

We had an unexpectedly low rate of postoperative delirium thus we were unable to determine if postoperative delirium was reduced in older adults with newly diagnosed OSA receiving APAP vs. those who did not receive APAP after elective knee or hip arthroplasty.

**Trial registration:**

This trial was retrospectively registered in clinicaltrials.gov NCT02954224 on 03/11/2016.

## Background

Globally, approximately 703 million people are 65 years of age or over, with the number expected to double to 1.5 billion in 2050 [1]. Delirium is a common postoperative complication with an incidence of up to 40% in older patients [2]. The incidence in elective orthopedic surgery is between 3.6% and 41% [3, 4]. Postoperative delirium is associated with longer hospital length of stay, increased mortality, and short- and long-term morbidity [4]

Obstructive sleep apnea (OSA) is a risk factor for postoperative complications [5–8] and several studies have reported an association between OSA and postoperative delirium [9–11]. In the general population, OSA is associated with neurocognitive impairment [12] and treatment with continuous positive airway pressure (CPAP) for 3 months [13] to one year [14] has been shown to improve cognition. Pre-existing cognitive impairment is a known risk factor for postoperative delirium [4]. Thus, patients with unrecognized or untreated OSA may be at greater risk for developing postoperative delirium [15]. To date, there is limited literature on the association between OSA and delirium in the surgical population. Conflicting results have been reported on whether OSA increases the risk of postoperative delirium [9–11, 16, 17] and whether treatment is protective [18].

The prevalence of moderate to severe OSA is 17% in 50–70 year old men and 9% in 50–70 year old women [19] however, OSA is frequently undiagnosed [20] as some of the symptoms may be attributed to normal aging [21]. Thus, OSA may be a modifiable risk factor which can be identified in the pre-operative assessment clinic and treated peri-operatively to reduce postoperative complications such as delirium. The prevalence of OSA is high in patients undergoing elective joint replacement; 29.3% patients had moderate to severe OSA and 7.3% had severe OSA [22]. The prevalence is increasing with the rise in obesity, which is also a risk factor for hip and knee osteoarthritis requiring arthroplasty surgery [23]. Our objectives were to identify OSA pre-operatively with a home sleep apnea test (HSAT) and to determine whether auto-titrating positive airway pressure (APAP) administered in the postoperative setting reduces the incidence of postoperative delirium in older adults undergoing elective hip or knee arthroplasty. We hypothesized that APAP would reduce the risk of postoperative delirium.

## Methods

The prevention of delirium in elderly with obstructive sleep apnea study (PODESA) was a multi-center, randomized controlled superiority trial conducted over three years in three academic centres in Toronto, Ontario, Canada (Toronto Western Hospital, Mount Sinai Hospital, and Sunnybrook Hospital). Ethics approval for this study was provided by Ethics Committees at Toronto Western Hospital (Approval no. 14–8710.0), Mount Sinai Hospital (Approval no. 15–0017-A), and Sunnybrook Hospital (Approval no. 113–2015). Written informed consent was obtained from all trial participants. This study was performed in accordance with the Declaration of Helsinki. We reported the trial objectives, design, and methods previously [24]. This study was registered at clinicaltrials.gov NCT02954224 on 03/11/2016 with JW as the Principal Investigator. This manuscript adheres to the applicable CONSORT guidelines.

### Participants

Eligible patients were aged 60 years or older scheduled for elective hip or knee arthroplasty greater than four working days after their pre-operative clinic appointment, and who were proficient in English (reading level grade 6 or above). They were required to be capable of completing the HSAT by themselves, study questionnaires, and be accessible for telephone follow-up postoperatively.

Exclusion criteria included: 1) schizophrenia, active psychosis within the previous 3 months, or current use of antipsychotic medication; 2) anxiety disorder, poorly controlled depression, or multiple psychiatric disorders; 3) history of drug or alcohol dependence or abuse within the last 3 months; 4) dementia or other clinically significant neurological disorders (e.g. stroke, epilepsy, brain tumour, Parkinson’s disease; 5) two-stage surgery or more than one surgical procedure within the same hospital admission; 6) central sleep apnea (greater than 50% central events or significant Cheyenne-Stokes pattern on sleep study); 7) significant cardiac disease (New York Heart Association functional class III or IV, severe valvular heart disease, dilated cardiomyopathy, pacemaker or implanted defibrillators, unstable angina), myocardial infarction, cardiac surgery or percutaneous coronary intervention within the previous 3 months; and 8) severe tracheal or lung disease, or other contraindication to non-invasive positive airway pressure (PAP) therapy. Although patients with a prior diagnosis of sleep-disordered breathing with treatment were excluded, those who had a previous sleep study but were either lost to follow-up, not established or non-adherent with treatment (less than 4 h per night or median less than 50% total sleep time continuous positive airway pressure [CPAP] or APAP use) were included in the study.

### Intervention

After screening in the pre-operative clinic, patients meeting the eligibility criteria and who consented were assessed for risk of sleep-disordered breathing using the STOP-Bang questionnaire [25–27] and Epworth Sleepiness Scale [28]. They also completed a Charlson comorbidity index [29] and Mini-Cog test [30, 31]. Patients with a STOP-Bang score of three or higher underwent a HSAT using the portable ApneaLink Air™, (ResMed, San Diego, California) a Type III sleep apnea monitoring device, which analyzes respiratory effort, heart rate, oxygen saturation, nasal airflow, and snoring. The sleep data were manually scored and checked by a qualified sleep technologist and interpreted by the study sleep physician (MS). If inadequate sleep or any technical failure occurred, the patient repeated the HSAT once. The conduct, data retrieval, and interpretation of the sleep study were consistent with the Canadian Sleep Society, [32] and American Academy of Sleep Medicine [33] guidelines for portable monitors. The scoring criteria for breathing events was consistent with the American Academy of Sleep Medicine definition [34]. An apnea was defined as a decrease in airflow by ≥ 90% of baseline for ≥ 10 s. A hypopnea was defined as a decrease in airflow by > 30% baseline for 10 s in association with either 3% oxygen desaturation from pre-event baseline and/or the event was associated with an arousal [34].

Patients identified as having OSA by HSAT (apnea hypopnea index [AHI] ≥ 10 events/hr) were randomized to the treatment (APAP) or control (routine care) group. APAP may be used when there is insufficient time to establish CPAP therapy guided by a sleep study. It alters the level of positive pressure delivered by the machine according to physiological variables such as snoring (vibration), airflow limitation or flow-versus-time [35]. Auto-titration is as effective for reducing the AHI as a fixed level of CPAP derived through polysomnography [35]. In the peri-operative setting, APAP may have advantages over CPAP due to rostral fluid shifts, changes in sleeping position, residual sedative effect of general anesthesia, and opioids that may make preoperative CPAP settings less effective after surgery [36, 37].

The treatment group received APAP using the AirSense™ 10 Autoset™ (ResMed, San Diego, California) device during periods of sleep for 72 h postoperatively or until discharged from hospital, whichever occurred sooner. If the patient was able to return to the pre-operative clinic before the scheduled surgery, APAP was provided to the patient to use one to three days before surgery to allow them to become accustomed to using it. Mask fitting and training was provided to the patient by a trained research assistant. The minimum pressure was set at 5 cm H20 and the maximum pressure was set at 20 cm H20. Since the severity of OSA may worsen postoperatively, [38] the wide range of APAP allowed for the optimal pressure to abolish OSA by titrating upwards to eliminate obstructive events. Data relating to APAP adherence, AHI, and other parameters were recorded on the AirView™ server (ResMed, San Diego, California). Supplemental oxygen was administered through a “Y” shape connector between the mask and the APAP device, at the discretion of the health care team if clinically indicated [38].

There were no changes to the usual peri-operative management of patients in both groups at the participating centers. A standardized anesthetic approach with spinal anesthesia, with a peripheral nerve block (knee arthroplasty), and intravenous sedation with midazolam 1–2 mg prior to the spinal and/or nerve block was used as per usual practice at the participating centers. Intraoperatively, sedation was provided with propofol 25–100 mcg/kg/min targeting a Ramsay Sedation Scale of 3–4. Multimodal postoperative analgesia with oral hydromorphone or morphine, acetaminophen, and/or cyclooxygenase-2 inhibitors were provided as per usual practice. The morphine milligram equivalents (MME) were calculated using the 2016 Center for Disease Control guidelines [39].

The routine care group did not receive APAP and were cared for according to standard practices provided by the pre- and postoperative care team. This may have included oxygen therapy, and any other interventions (including CPAP) deemed necessary by the treating physicians.

### Outcomes

Patients were assessed for the primary outcome; presence of postoperative delirium by trained research assistants beginning on the night of surgery (considered postoperative night one [N1]), and then twice daily until 72 h postoperatively or discharge, whichever was earlier. The 3D Confusion Assessment Method (CAM) was used to identify delirium [40]. The first postoperative assessment was performed when the patient reached a Richmond Agitation Sedation Score of -3 or higher, indicating that they were sufficiently recovered from the sedative effects of anesthesia to be adequately assessed for delirium using the CAM. A minimum of 6 h occurred between assessments. Patients who remained delirious more than 72 h postoperatively were followed up by the research assistant with twice-daily CAM assessment until resolution of symptoms, defined as being CAM-negative. We considered the patient to have delirium if the following features were present: 1) acute onset or fluctuating course and 2) inattention and 3) either disorganized thinking or 4) altered level of consciousness. If any item of the 4 features was answered incorrectly, the patient was considered to be positive for that feature. Patients’ hospital charts were also examined for evidence of delirium [41]. Patients were considered to have delirium if at least one CAM assessment was positive or if delirium was documented in their hospital chart.

Secondary outcomes were length of inpatient stay (LOS), and postoperative complications (including intensive care unit (ICU) admission, oxygen desaturation (SpO2) < 90%, re-intubation, myocardial infarction, surgical site infection, deep vein thrombosis, pulmonary embolus, pneumonia, urinary tract infection, sepsis, stroke) occurring up to 30 days postoperatively.

Oximetry monitoring with a high-resolution pulse oximeter wristwatch (PULSOX-300i, Konica Minolta Sensing, Inc., Osaka, Japan) was carried out for one night before surgery and for three nights postoperatively or until discharge if the hospital stay was < 72 h. The oximeter was applied to the non-dominant hand.

### Sample size

With an expected incidence of postoperative delirium of 24% in patients with untreated OSA, [9, 10] 220 patients (110 in each group) gave 80% power at α = 0.05 to show a 58% relative risk reduction to incidence of 10% with APAP treatment. In a previous study, the incidence of OSA was 45.9%, [38] therefore we expected 480 patients needed to be screened to identify 220 patients with OSA. Assuming an attrition rate (due to study withdrawal or test failure) of 10%, we estimated that 528 patients needed to be recruited into the study.

### Randomization

A computer-generated randomization list with block sizes of 8 for each participating site, in a 1:1 ratio of APAP to routine care, was created by a research analyst not involved in the study. Patient assignments were placed in serially numbered, opaque, sealed envelopes, and only opened by the research assistant at each site after the diagnosis of OSA was confirmed by HSAT.

### Blinding

It was not possible to blind the patients or care team to the treatment using a placebo device as the APAP machine could not be programmed to deliver zero cm H2O. Study investigators and the statistician involved in data analysis were blinded to the intervention.

### Statistical analysis

The primary outcome: presence of postoperative delirium was compared between the APAP and routine care group using the Fisher exact test and a 95% confidence interval (CI) score-test based CI for the difference in incidence [42]. Modified intention-to treat analysis was performed for the primary and secondary outcomes, including only those who received the allocated intervention. Continuous secondary outcomes were summarized by the mean and standard deviation and compared between groups using the t-test or by the median and interquartile range (IQR) and compared between groups using the Wilcoxon rank sum test. Binary outcomes were summarized as counts and proportions and compared between groups using Chi-squared tests or Fisher’s exact tests. Comparisons with a *p*-value < 0.05 were considered statistically significant. All statistical analyses were performed using R 4.03 [43].

## Results

**Fig. 1:**
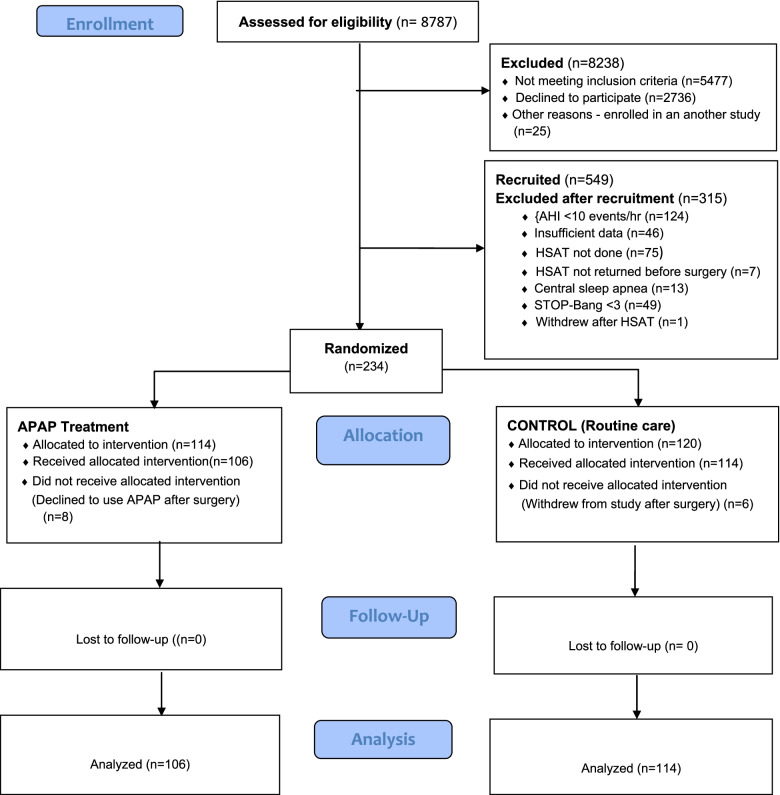
Flow diagram of patient enrolment and follow-up. APAP: auto-titrating positive airway pressure, HSAT: home sleep apnea test, AHI = apnea hypopnea index

Recruitment occurred from March 2016 to June 2019. Of the 8,787 patients assessed for eligibility, 8,238 patients were excluded for various reasons (Fig. 1). Of the 549 patients recruited, 474 completed the HSAT. A total of 240 patients were excluded after the HSAT due to: AHI < 10 events per hour (*n* = 124), insufficient HSAT data (*n* = 46), technical issues, or other reasons (Fig. 1). Thus, 234 patients were randomized to APAP treatment (114 patients) or control – routine care (120 patients). Eight patients in the APAP arm and 6 patients in the routine care arm discontinued study participation after surgery. Analysis was performed in 220 patients (Fig. 1).

The demographic characteristics of the patients are shown in Table 1. Ten (4.5%) patients who completed the Mini-Cog before surgery, screened positive for mild cognitive impairment (Mini-Cog score ≤ 2) (Table 1). Thirty-three percent of patients had mild OSA, 41.8% had moderate OSA, and 25% had severe OSA (Table 2). The preoperative baseline oxygen desaturation index (ODI), average and minimum oxygen saturation, cumulative time oxygen saturation less than 90% (CT90) were similar between the two groups (Table 2).

**Table 1:** Baseline characteristics

	Overall	APAP	Routine
n	220	106	114
Age (mean (SD))	68.2 (6.2)	67.8 (5.7)	68.5 (6.7)
Male Gender, n (%)	129 (58.6)	60 (56.6)	69 (60.5)
Weight kg (mean (SD))	95.8 (20.2)	96.0 (19.5)	95.7 (20.9)
BMI (mean (SD))	33.3 (6.3)	33.3 (6.1)	33.4 (6.4)
ASA Status, n (%)			
1	4 ( 1.8)	1 ( 0.9)	3 ( 2.6)
2	100 (45.5)	48 (45.3)	52 (45.6)
3	112 (50.9)	54 (50.9)	58 (50.9)
4	2 ( 0.9)	2 ( 1.9)	0 ( 0.0)
not recorded	2 ( 0.9)	1 ( 0.9)	1 ( 0.9)
Charlson co-morbidity index (mean (SD))	2.6 (0.9)	2.6 (1.00)	2.6 (0.8)
Hypertension, n (%)	140 (63.6)	70 (66.0)	70 (61.4)
Congestive Heart Failure, n (%)	3 ( 1.4)	2 ( 1.9)	1 ( 0.9)
Prior Myocardial Infarction, n (%)	8 ( 3.6)	4 ( 3.8)	4 ( 3.5)
Smoker, n (%)	49 (22.3)	24 (22.6)	25 (21.9)
Diabetes, n (%)	32 (14.5)	16 (15.1)	16 (14.0)
STOP-Bang Score (mean (SD))	5.0 (1.3)	5.0 (1.4)	5.0(1.2)
ESS Score (mean (SD))	6.2 (4.2)	6.7 (4.2)	5.8 (4.1)
Knee Surgery, n (%)	139 (63.2)	68 (64.2)	71 (62.3)
MiniCog Score, n (%)			
≤ 2	10 ( 4.5)	4 ( 3.8)	6 ( 5.3)
> 2	177 (80.5)	87 (82.1)	90 (78.9)
Missing	33 (15.0)	15 (14.2)	18 (15.8)

*APAP* auto-titrating positive airway pressure, *SD* standard deviation, *SMD* standardized mean difference, *BMI* body mass index, *ASA* American Society of Anesthesiologists, *ESS* Epworth Sleepiness Scale

**Table 2:** Baseline oximetry and OSA screening

	Overall	APAP	Routine
n	220	106	114
CT90 (median, [IQR])	4.8 [1.4, 15.1]	4.4 [1.5, 12.8]	5.2 [1.1, 15.3]
Min. SpO2 (mean, SD)	75.9 (10.8)	75.2 (12.6)	76.6 (8.7)
Average SpO2 (mean, SD))	92.5 (2.1)	92.5 (2.4)	92.5 (1.8)
ODI (events/hr) (median [IQR])	19.3 [11.4, 28.0]	19.6 [12.2, 28.0]	18.7 [11.1, 28.2]
Pre-op. diagnosis and treatment of OSA (n, %)			
No	209 (95.0)	99 (93.4)	110 (96.5)
Yes—untreated	11 ( 5.0)	7 ( 6.6)	4 ( 3.5)
Yes—treated	0 ( 0.0)	0 ( 0.0)	0 ( 0.0)
Screening AHI (n, %)			
5–14.9 (mild)	73 (33.2)	33 (31.1)	40 (35.1)
15–29.9 (moderate)	92 (41.8)	46 (43.4)	46 (40.4)
≥ 30 (severe)	55 (25.0)	27 (25.5)	28 (24.6)

*APAP* auto-titrating positive airway pressure, *CT90* cumulative time oxygen saturation less than 90%, *IQR* interquartile range, *SpO2* oxygen saturation, *SD* standard deviation, *OSA* obstructive sleep apnea, *AHI* apnea hypopnea index

### Delirium and postoperative outcomes

In total, 2.7% (6/220) patients developed delirium after surgery: 4.4% (5/114) patients in the routine care group, and 0.9% (1/106) patients in the APAP group (risk reduction 3.4%; 95% CI: -1.1% to 8.7%; *P* = 0.21). The patient in the APAP group who developed postoperative delirium was APAP non-adherent. One patient developed delirium on the night of surgery, two on postoperative day one (D1), and three on D2. One patient had delirium that persisted for two days until D3. Of the six patients who developed delirium, three were found to have cognitive impairment on the Mini-Cog and three did not complete the Mini-Cog before surgery. None of the 124 patients who had an AHI < 10 events per hour developed delirium. The median and interquartile range for LOS were identical for the APAP vs. routine care group at 2 days (IQR 2–3). The mean (SD) LOS was not significantly different between APAP vs routine care (2.9 (2.9) days vs 3.5 (4.4) days, *p* = 0.28). There was no difference in duration of surgery, intraoperative sedation, intraoperative or postoperative complications on the ward or at 30 days between the two groups. One patient in each group had an unplanned ICU admission. The MME were similar between the two groups on postoperative D1 to D3. The MME for the APAP vs. routine care group were 116.7(140.6) vs. 90.4 (92.7), 52.7 (53.1) vs. 53.3 (54.4), and 21.1 (38.9) vs. 17.2 (30.3) on postoperative D1, D2, and D3 respectively.

### Adherence with APAP

**Fig. 2:**
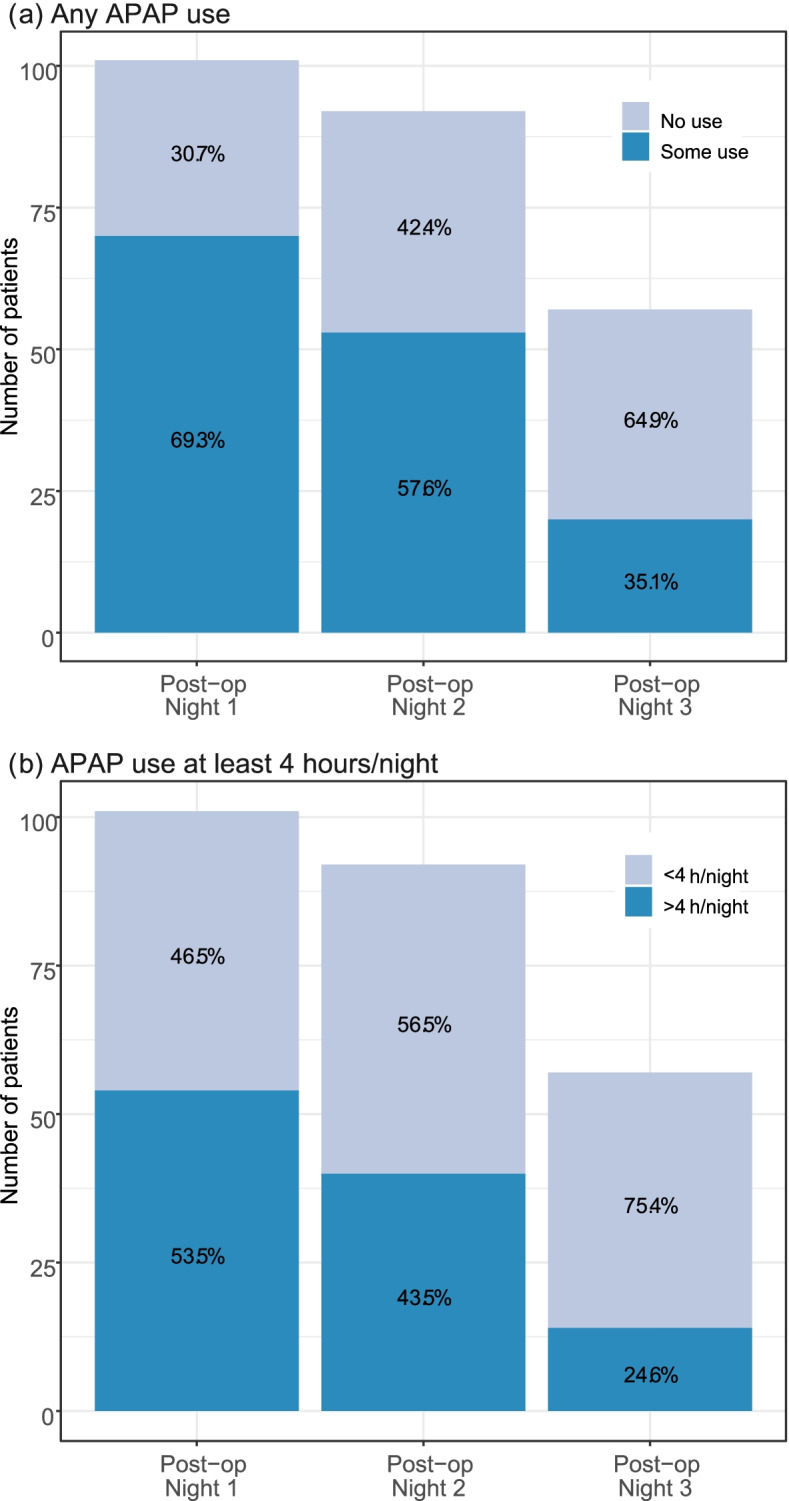
Adherence with postoperative auto-titrating positive airway pressure therapy. 2a. Any APAP use 2b. APAP use of at least 4 h/night. APAP: auto-titrating positive airway pressure

Thirty-nine patients were given APAP before surgery. For the remaining patients who were randomized to APAP, there was either insufficient time or they were unable to return to hospital to obtain the APAP machine prior to surgery. In the APAP group, 84.5% (87/106) of patients used APAP postoperatively. The median and interquartile range of APAP use was 4.4 (0.8–7.4) hours, 2.4 (0–6.4) hours, and 0.1 (0–3.8) hours on postoperative N1 to N3, respectively. A total of 69.3% of patients were APAP adherent on postoperative N1, 57.6% on N2, and 35.1% on N3 (Fig. 2a). On postoperative N1, 53.5% of patients used APAP for 4 h/night or more, this decreased to 43.5% on N2, and 24.6% on N3 (Fig. 2b).

None of the patients in the control group were prescribed CPAP.

### Postoperative oximetry and supplemental oxygen

The mean average SpO2 was similar on post-operative N1-3 in the APAP and routine care groups (Table 3). On postoperative N1 and N2, there was no difference in the lowest SpO2 between the two groups (Table 3). However, on N3, the lowest SpO2 was higher in the APAP compared to the routine care group (76.1 (7.1) vs. 69.6 (11.1) %, *P* = 0.008). The ODI and CT90 were similar between the two groups on postoperative N1-3 (Table 3). When comparing the APAP vs. routine care groups, supplemental oxygen was administered to 44.6% vs. 49.5% of patients on N1, (*P* = 0.553); 15.9% vs. 9.9% on N2, (*P* = 0.335), and 6.7% vs. 5.4% on N3, (*P* = 1.0). (Table 3).

**Table 3:** Postoperative oximetry and use of supplemental oxygen

	APAP	Routine	p
**Post-operative night 1**			
n	83	99	
CT90 (median [IQR])	5.1 [0.7, 27.9]	3.3 [0.6, 18.4]	0.336
Min SpO2 (mean (SD))	75.4 (11.0)	74.7 (11.2)	0.647
Average SpO2 (mean (SD))	92.1 (3.8)	93.1 (3.7)	0.070
ODI (events/hr) (median [IQR])	17.8 [8.9, 26.5]	13.9 [6.4, 24.6]	0.318
Supplemental oxygen (n, %)	37 (44.6)	49 (49.5)	0.553
**Post-operative night 2**			
n	69	91	
CT90 (median [IQR])	7.9 [2.0, 35.5]	19.0 [3.0, 48.0]	0.158
Min SpO2 (mean (SD))	73.6 (13.3)	72.5 (11.2)	0.564
Average SpO2 (mean (SD))	91.2 (5.2)	90.5 (4.7)	0.372
ODI (events/hr) (median [IQR])	20.3 [9.9, 31.0]	21.5 [14.2, 38.6]	0.210
Supplemental oxygen (n, %)	11 (15.9)	9 (9.9)	0.335
**Post-operative night 3**			
n	30	37	
CT90 (median [IQR])	13.8 [5.2, 42.8]	21.1 [4.2, 61.7]	0.623
Min SpO2 (mean (SD))	76.1 (7.1)	69.6 (11.1)	0.008
Average SpO2 (mean (SD))	90.5 (4.0)	90.7 (2.9)	0.790
ODI (events/hr) (median [IQR])	20.7 [12.5, 40.8]	20.8 [12.5, 42.1]	0.703
Supplemental oxygen (n, %)	2 (6.7)	2 (5.4)	1.000

*APAP* auto-titrating positive airway pressure, *CT90* cumulative time oxygen saturation less than 90%, *IQR* interquartile range, *SpO2* oxygen saturation, *SD* standard deviation, *ODI* oxygen desaturation index

## Discussion

Our study investigated the effect of APAP on delirium in older adult surgical patients with newly diagnosed OSA confirmed with HSAT. We did not find a lower incidence of postoperative delirium in the APAP group (0.9%) compared to the routine care group (4.4%). We were unable to show the expected relative risk reduction of delirium due to the incidence of delirium being lower than reported in previous literature.

An increasing number of studies have shown an association between OSA and increased risk for cognitive decline and Alzheimer’s disease [44–46]. Mild cognitive impairment was found in 47.9% patients with suspected OSA referred to academic sleep centers [47]. An association between OSA and postoperative delirium has been reported in several studies [9–11]. A previous study of patients undergoing elective total knee arthroplasty reported a possible association between previously diagnosed OSA and increased risk of postoperative delirium [9]. Another study found a sixfold increase in postoperative delirium in cardiac surgical patients with sleep-disordered breathing [11]. As well, a preliminary analysis of an observational cohort study of 66 patients found a relationship between high STOP-Bang scores and cognitive decline 6 weeks after surgery [48].

In a randomized controlled trial (RCT) of 114 patients at risk for OSA (STOP-Bang score of three or higher) undergoing elective knee or hip arthroplasty, pre-operative apnea severity had a significant association with delirium severity [18]. Similar to our study, they did not show a reduction in delirium with PAP. However, the adherence with postoperative CPAP treatment was low with a median use of one night, and only 1.4 h on postoperative N1 [18]. Another RCT of postoperative APAP in 86 patients at high risk for OSA found no significant difference in LOS or complications, including delirium [49] However, they did not reach their planned sample size and the rate of complications was low. Adherence with postoperative PAP therapy in patients with newly diagnosed OSA is challenging. A previous study reported 26–48% of patients used APAP for 4 h/night [38]. Unfortunately, in our study, many patients were unable to become accustomed to APAP prior to surgery because there was insufficient time between return of the HSAT and planned surgery. CPAP must be used consistently for at least 4 h per night for a therapeutic response [50]. Although almost 70% patients used APAP on the first night, only about half of the patients used it for at least 4 h and the adherence decreased on subsequent nights.

Long-term adherence with CPAP was associated with maintenance of cognitive performance in older adults with severe OSA who were followed over 3 years [51]. Treatment with CPAP for three months was associated with improvements in quality of life and some neurocognitive functions in adults ≥ 70 with severe OSA [13]. Another study of older adults with mild cognitive impairment and OSA showed a significant improvement in cognition (improved psychomotor/cognitive processing speed), daytime sleepiness, and daily function in the CPAP adherent group (4 h per night use) versus the CPAP non-adherent group a year after their OSA diagnosis [14].

In contrast, a recent observational study of over 1400 patients failed to show an independent association between OSA and postoperative delirium [16]. However this study included patients at high risk of OSA by STOP-Bang score rather than diagnosed by sleep study and the actual adherence and number of hours of postoperative CPAP usage were not reported [16]. Another recent retrospective study of non-neurosurgical patients admitted to ICU reported no association between OSA risk and postoperative delirium [17]. Similar to Strutz et al.’s study, the OSA diagnosis was either self-reported or based on STOP-BANG questionnaire scores. Although questionnaires are helpful as a screening tool to predict risk for OSA, questionnaires are not as accurate for diagnosis compared to sleep studies and may have higher false positive results [52].

Our incidence of delirium was lower than reported previously in the literature. Due to the low incidence of postoperative delirium, our study is underpowered to detect a difference in use of APAP to prevent postoperative delirium. The low rate of delirium in our study may be due to the use of enhanced recovery procedures including improvements in surgical techniques, the use of regional anesthesia with minimal midazolam, multimodal non-opioid postoperative analgesia, early mobilization and early discharge from hospital. Although the CAM assessments were conducted twice a day; it is possible that patients may have experienced delirium in between the CAM assessments. Our incidence of postoperative delirium is similar to a study with over 1.6 million patients which found an incidence of postoperative delirium of 2.6% in patients undergoing elective hip and 2.9% in elective knee arthroplasty [53]. A much larger study would be needed to determine whether APAP has a plausible treatment effect on delirium.

The low rate of cognitive impairment in our study (5.3%) contrasts with a recent systematic review and meta-analysis that showed older adults undergoing elective orthopedic surgery had a pooled prevalence of unrecognized cognitive impairment of 37% (95% CI 26–49%) [54]. This suggests there may be participant bias as participants in our study had to have the cognitive and physical capability to properly do the HSAT without assistance. It was impractical for a research assistant to go to the patient’s home to assist them with the HSAT. Older patients undergoing knee or hip replacement who screened positive for probable cognitive impairment on the Mini-Cog have previously been shown to have a 4.5-fold greater odds of developing delirium [55]. Thus, the patients in our study may have been at lower risk for developing postoperative delirium since pre-existing cognitive impairment is known to be a major risk factor for delirium.

APAP did not improve the ODI, average SpO2, minimum SpO2 or CT90 on postoperative N1 and N2. A possible reason for this finding may be due to only about half of the patients using APAP for at least 4 h on postoperative N1, and declining adherence on subsequent nights. As well, the administration of supplemental oxygen to a significant number of patients in both groups on postoperative N1 may have prevented/masked hypoxemia in patients in the routine care group. The use of supplemental oxygen progressively decreased over time. On N3, the lowest SpO2 was significantly lower in the routine care compared to the APAP group, likely because only 1.8% of patients in the routine care group received supplemental oxygen by N3.

### Limitations

There are a few limitations of this study. Many patients were unable to use APAP prior to surgery because they were not able to return to the hospital to obtain the APAP or there was insufficient time between return of HSAT and planned surgery. APAP adherence may have been higher if patients were able to become accustomed to using APAP before surgery. Second, the research assistant performing the delirium assessment was not blinded to the treatment group since APAP cannot be set to zero cm H2O. Finally, the incidence of delirium was lower than previously reported in the literature, and a larger study would be needed to show a plausible treatment effect.

## Conclusions

Due to the unexpectedly low incidence of postoperative delirium in this study we were unable to demonstrate a benefit of APAP. Future studies should include a large sample size and consider a population with a higher incidence of delirium. As well, starting APAP prior to surgery rather than after surgery may allow patients to become accustomed to using APAP and increase adherence.

## Data Availability

The datasets generated and/or analysed during the current study are not publicly available but are available from the corresponding author on reasonable request.

## Abbreviations

AHI: Apnea hypopnea index
APAP: Auto-titrating positive airway pressure
CAM: Confusion Assessment Method
HSAT: Home sleep apnea test
ICU: Intensive care unit
LOS: Length of stay
OSA: Obstructive sleep apnea
PAP: Positive airway pressure
PODESA: Prevention of delirium in elderly surgical patients with obstructive sleep apnea
RCT: Randomized controlled trial
